# Reduction of CMOS Image Sensor Read Noise to Enable Photon Counting

**DOI:** 10.3390/s16040517

**Published:** 2016-04-09

**Authors:** Michael Guidash, Jiaju Ma, Thomas Vogelsang, Jay Endsley

**Affiliations:** 1 Rambus Inc., Sunnyvale, CA 94089, USA; tvogelsang@rambus.com (T.V.); jendsley@rambus.com (J.E.); 2 Thayer School of Engineering, Dartmouth College, Hanover, NH 03755, USA; jiaju.ma@dartmouth.edu

**Keywords:** CMOS, image sensor, photon counting, read noise

## Abstract

Recent activity in photon counting CMOS image sensors (CIS) has been directed to reduction of read noise. Many approaches and methods have been reported. This work is focused on providing sub 1 e^−^ read noise by design and operation of the binary and small signal readout of photon counting CIS. Compensation of transfer gate feed-through was used to provide substantially reduced CDS time and source follower (SF) bandwidth. SF read noise was reduced by a factor of 3 with this method. This method can be applied broadly to CIS devices to reduce the read noise for small signals to enable use as a photon counting sensor.

## 1. Introduction

### 1.1. Read Noise Reduction for CIS Devices

In the past several years there has been a substantial amount of work directed to the use of CMOS Image Sensors (CIS) for single photon detection and photon counting [1,2,3,4,5]. A key requirement and development area for photon counting CIS devices is low read noise [1,2,3,4,5]. It has been shown that CIS read noise should be reduced to 0.15 electrons (e^−^) or less [2]. The state of the art for high volume consumer application small pixel CIS is in the 1.2 e^−^ to 2.0 e^−^ range. Recent work on CIS read noise reduction has been directed to increasing conversion gain (CG) [6,7,8,9], correlated multiple sampling (CMS) [9,10], source follower (SF) transistor structure [11], and SF accumulation [12]. Results of these papers are summarized in Table 1. Sub 1 e^−^ rms read noise was achieved with results in the range of 0.28 e^−^ rms to 0.86 e^−^ rms.

### 1.2. New Method for Read Noise Reduction for Photon Counting CIS Devices

This paper addresses a different approach to read noise reduction for photon counting CIS devices. For single bit photon counting CIS devices, the pixel output signal readout will be binary (*i.e.*, no signal or >1 photon signal) [1]. In this case the signal readout path needs to be designed for a signal range that corresponds to 1 e^−^ with some headroom (e.g., 5 e^−^). There is no reason to measure or precisely know the output signal value above this maximum signal. For conversion gains in the range of 200 μV/e^−^ to 500 μV/e^−^ this is a maximum signal swing of 1–2.5 mV. For multi-bit photon counting CIS devices the pixel output signal swing needs to be precisely known only for signal levels corresponding to the maximum number of electrons to be counted per readout (e.g., 20 e^−^) [2,3]. As a result the maximum output signal to be precisely determined is <10 mV. In both cases this is substantially less than the maximum signal swing for a conventional CIS device, which is typically on the order of 0.5–1.0 V. The signal readout path can be designed and optimized for this. Since the maximum signal level that needs to be accurately quantified is small compared to a conventional CIS, a shorter Correlated Double Sample (CDS) time (*t*_CDS_) and reduced source follower (SF) bandwidth (BW) can be used for a photon counting CIS compared to a conventional CIS.

**Figure 1:**
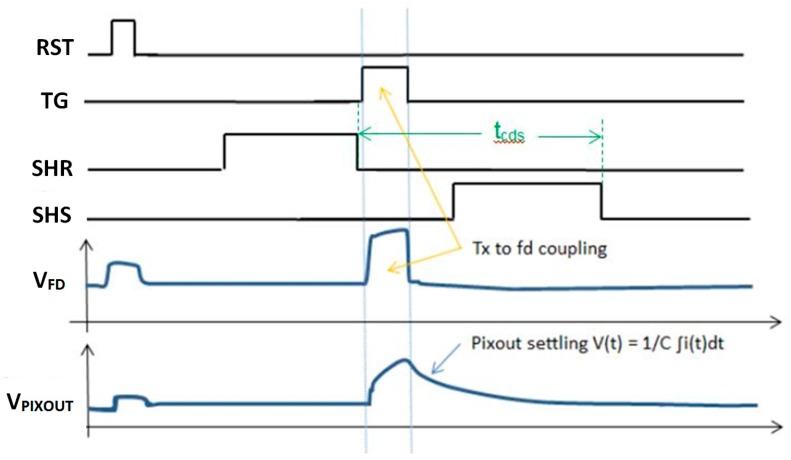
Conventional pixel readout timing diagram and output waveform. TG feed-through limits small signal settling time.

Referring to Figure 1, the *t*_CDS_ is defined as the time between the falling edge of sample-and-hold reset (SHR) pulse to the falling edge of sample-and-hold signal (SHS) pulse. SF read noise is limited by 1/f noise and Random Telegraph Signal (RTS) noise, [13,14]. Reduction of *t*_CDS_ and SF BW will have an attendant reduction on 1/f and Johnson or thermal noise of the SF readout [13,15]. However, it is difficult to achieve significantly reduced *t*_CDS_ and SF BW due to the limitations of transfer gate (TG) feed-through (FT) to the floating diffusion (FD). This is shown in Figure 1.

**Figure 2:**
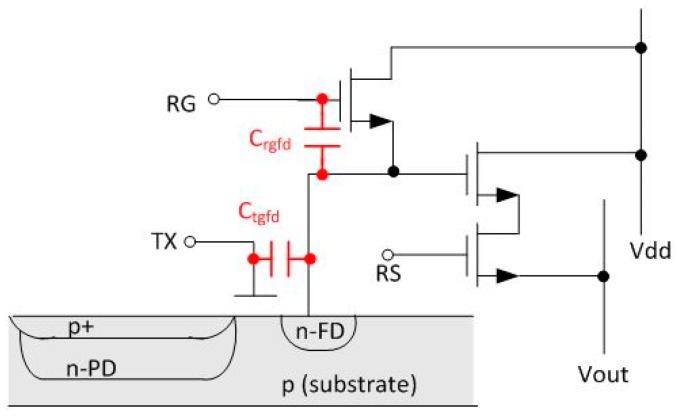
Pixel schematic with TG and RG coupling capacitances to FD.

The coupling capacitance from TG to FD (*C*_tgfd_) and reset gate (RG) to FD (*C*_tgrg_) is shown in Figure 2. The FD node will see a FT signal that follows the pulses from TG and RG signals. The magnitude of the FT signal (Δ*V*_FT_) is given by:

(1) $$\Delta V_{\text{FT}}=\Delta V_{\text{tg}}*\left(C_{\text{tgfd}}/C_{\text{fd}}\right)$$

where Δ*V*_tg_ is the voltage swing of the TG pulse and *C*_fd_ is the total capacitance of the FD node.

The pixel output settling time is dominated by the TG FT to the FD for small signal levels. This is especially true for high conversion gain pixels where the *C*_tgfd_ can be a larger percentage of *C*_fd_.

### 1.3. A New Timing Method for CIS Read Noise Reduction

**Figure 3:**
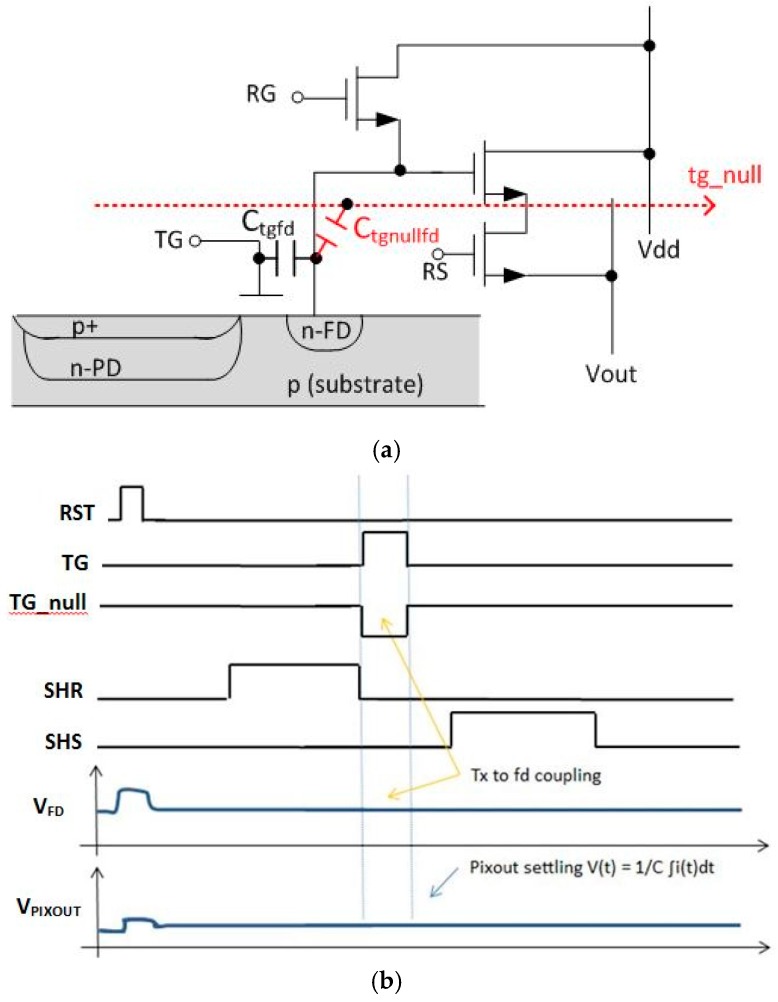
(**a**) New pixel schematic showing tg_null signal line used to compensate TG feed-through. (**b**) New pixel readout timing diagram and output waveform; TG feed-through is compensated.

In order to substantially reduce the *t*_CDS_ and SF BW we have devised a new readout timing method where TG feed-through is compensated and the small signal settling time is dramatically reduced. This is shown at a high level in Figure 3a,b below. A signal tg_null is used to null (*i.e.*, cancel or compensate) the TG feed-through. This signal can be provided as an additional and separate signal wire with an attendant decoder/driver. This null signal line is preferably row based, to match skew and droop over the array of the TG signals that are to be compensated. There are many possible approaches to provide the tg_null signal including use of existing pixel signal lines. One such approach is described in Section 2 of this paper.

Figure 3a is a pixel schematic showing the case of an additional row based signal line “tg_null”. There is a coupling capacitance from the tg_null signal line to the FD, *C*_tg_nullfd_. The magnitude of the nulling pulse designed to cancel the TG feed-through will depend on the value of *C*_tg_nullfd_. The compensation or feed-through cancellation signal does not have to be perfectly aligned with the TG signal in order to provide a substantially reduced pixel output settling time. For example, when referring to Figure 3b, the edges of the tg_null signal do not have to be exactly aligned with the TG signal pulse. In addition the product of the voltage swing and coupling capacitance (*C*_tg_nullfd_), of the tg_null pulse does not need to be exactly the same as that of the TG signal.

One of the advantageous effects of the feed-through compensation is the elimination of the trade-off between conversion gain (CG) and settling time. CG is the conversion factor of e^−^ to volts in the readout of the pixel. This is determined by *C*_fd_ according to Equation (2):

(2) $$CG=q/C_{fd}$$

If the feed-through to the floating diffusion is not compensated, the Δ*V*_FT_ will increase as the conversion gain is increased (*i.e.*, conversion gain is increased by decreasing *C*_fd_, so if coupling capacitance remains the same, the Δ*V*_FT_ is larger). This larger feed-through then causes a longer FD and *V*_out_ settling time and increases *t*_CDS_. By compensating the FD feed-through, one can increase CG without increasing settling time, and thus further reduce input referred read noise.

As mentioned above, an alternate approach to use of an additional null signal and signal line is to use existing signals and structures in the pixel for TG to FD feed-through signal compensation. This does not add capacitance to the FD and as a result does not reduce CG. One such approach was used on an existing sensor. The details of this method and attendant results are described in the next sections.

## 2. Materials and Methods 

### 2.1. Sensor Description

**Figure 4:**
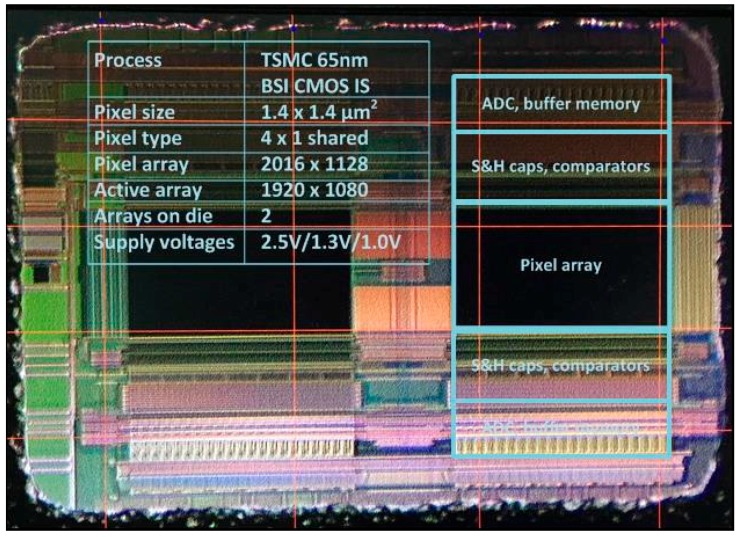
Sensor die photograph.

TG to FD feed-through compensation timing was implemented on an existing prototype sensor with programmable timing to investigate the effect of reduced *t*_CDS_ and SF BW on the sensor read noise. The chip photograph is shown in Figure 4.

**Figure 5:**
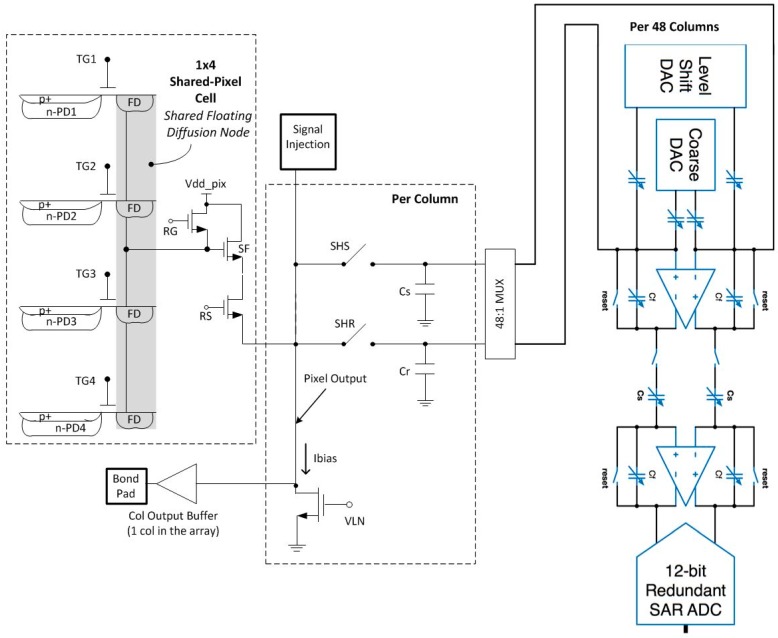
Block diagram of pixel array and readout signal path.

The chip contains two 1920 by 1080 pixel arrays. Each array contains different pixel architectures. One half of one the arrays is a 4-shared amplifier 4T pinned photodiode pixel architecture. The pixel size is 1.4 μm. The chip was fabricated in 65 nm BSI CIS process technology. A simplified schematic of the 4-shared unit pixel cell and the array readout path is shown in Figure 5.

The unit pixel cell is one column by four rows (1 × 4). The SF has a width of 0.28 μm and a length of 0.7 μm. The row select transistor has a width of 0.28 μm and a length of 0.29 μm. Per column sample and hold capacitors are used to store the reset and transfer signal levels for CDS readout. A switched capacitor programmable gain amplifier (PGA) and 12 bit SAR ADC are shared by 48 columns. The PGA and ADC layout is split into two banks, one at the top and one at the bottom of the array. Adjacent groups of four columns are routed to the top and bottom ADC banks. This architecture was chosen for fast readout. The PGA has a selectable gain of 2×, 4× or 8×. A gain of 8× was used for the noise measurements. One column output line is connected to an analog output buffer to view the pixel output waveform. An injection point was included at the input of the PGA to determine the electrons per Data Number (DN) of the readout path. The SF Ibias current is programmable by an external master current and on-chip current mirror. The sensor readout timing and control is implemented on a FPGA external to the sensor, and is fully programmable.

The pixel output lines have a total resistance of 1261 Ω and capacitance of 906 fF. The sample and hold capacitors are ~400 fF. The total capacitance (*C*_pixout_) of the column readout is ~1.3 pF (400 fF + 906 fF). The pixel output bandwidth is limited by the transconductance (*g*_m_) of the pixel source follower which is 12 to 55 μs depending on the SF bias current used in this experiment. The dominant time constant due to *g*_m_ (*τ*D = *C*_pixout_/*g*_m_), is ~22 ns at the baseline SF Ibias condition of 8 μA. The conversion gain of the 1 × 4 pixel is 75.6 μV/e^−^.

### 2.2. New Readout Method Details for Reduction of Read Noise

**Figure 6:**
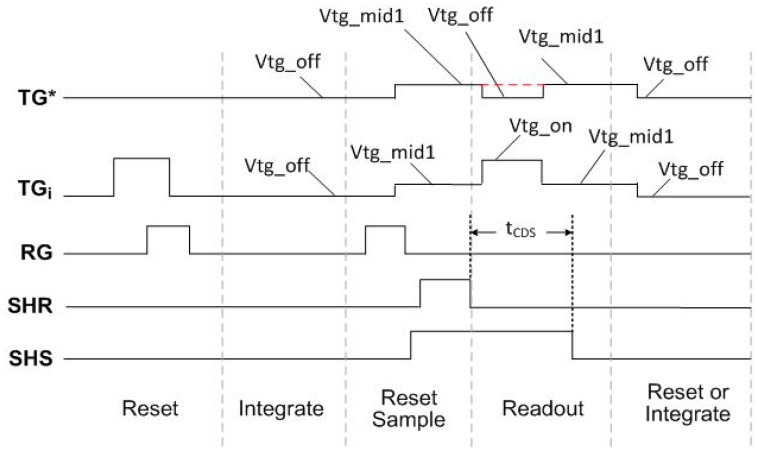
Intended pixel timing diagram for TG feed-through compensation: TGi is the row being readout; TG* is 3 “other” TG’s in the 1 × 4 pixel cell.

As discussed in the Introduction section, in conventional CIS timing and readout, the *t*_CDS_, is limited by the TG FT settling time, especially for small signals. In addition, for photon counting CIS devices, the *t*_CDS_ and SF BW can be reduced given the maximum output signal swing to accurately measure is very small compared to that of a conventional CIS device. We have modified the CIS timing to compensate or cancel the TGFT. By canceling the TGFT, the output signal settling time is reduced. A variety of timing approaches can be implemented depending on the pixel architecture and the row decoder/driver design details. The timing we intended to use is shown in Figure 6. The *t*_CDS_ is the time between the falling edge of the SHR signal pulse to the falling edge of the SHS signal pulse during the readout phase. The various TG signal levels are indicated by name. Vtg_off is the TG off level used during integration, and is typically a negative voltage in order to reduce TG dark current. Vtg_mid1 level is typically used during readout of the pixel, and is less negative or 0 V in order to avoid any gate induced drain leakage (GIDL) on the FD during readout. Vtg_on is the signal level used to provide lag free transfer from the photodiode (PD) to FD.

**Figure 7:**
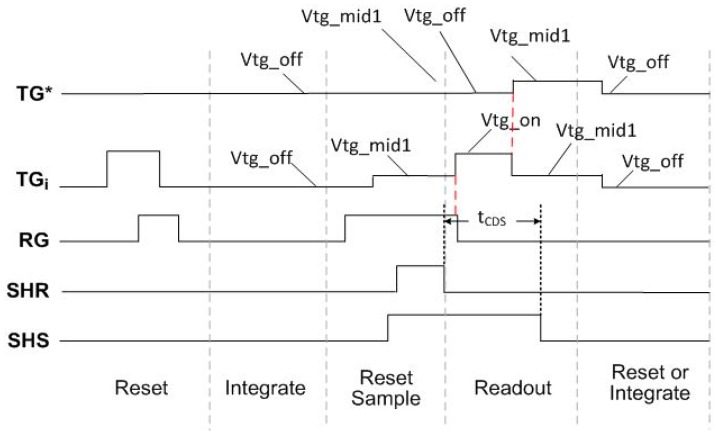
Pixel timing diagram used in this experiment due to limitations with sensor row decoder design.

Referring to Figure 6, for any given row being read out in the 1 × 4 unit cell, the other 3 TG’s are used to compensate the TG FT for the pixel being read out. With this approach, the compensating voltage of the three TG’s is in a sub-threshold range and will not cause charge transfer. Conventional TG timing is shown by the dotted red line for the TG* signal. Since the local overlap capacitance of the TGs to FD is well matched, this method will provide a very small residual FT signal, and the timing skew across the array will be very well matched for the TG* and TGi signals. Based on the row decoder/driver design of our sensor, we had to use the RG signal to compensate the rising edge of TG, and 3 TG’s to compensate the falling TG edge. This timing diagram is shown in Figure 7.

### 2.3. New Readout Method Measured Timing and Waveforms

**Figure 8:**
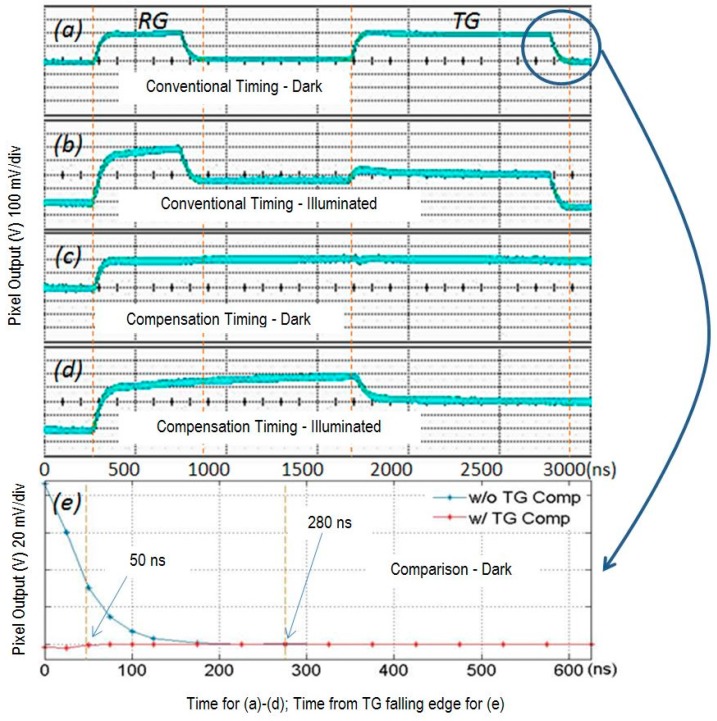
Analog output waveforms *vs.* time from TG falling edge.

Pixel output waveforms were captured at the column analog output buffer to verify operation of the timing and cancellation of the TG FT. These waveforms are shown in Figure 8.

Pixel output waveforms for conventional timing and our new timing are shown for both dark and illuminated conditions. The outputs for conventional timing for dark and illuminated conditions are shown in waveforms (a) and (b). The outputs for TG compensation timing for dark and illuminated conditions are shown in waveforms (c) and (d). The signal level for the illuminated condition is ~64 mV (~1000 e^−^). Comparing waveforms (a) and (c), it is evident that the compensation signals cancel the TG FT and the output settles much faster for both dark and illuminated signals. The zoomed in section of dark condition waveforms for conventional and compensation timing is shown in plot (e). This shows the dark level settling time is reduced from 280 ns to 50 ns by the TG FT compensation method. Note also that the RG falling edge feed-through is compensated by the TG rising edge. We briefly examined the variation in the residual feed-through for the single column of pixels that could be observed. The variation was very small, and we attribute this to the local matching of *C*_tgfd_ using this cancellation method. Further work is required to quantify this variation for the whole column and for an array.

**Figure 9:**
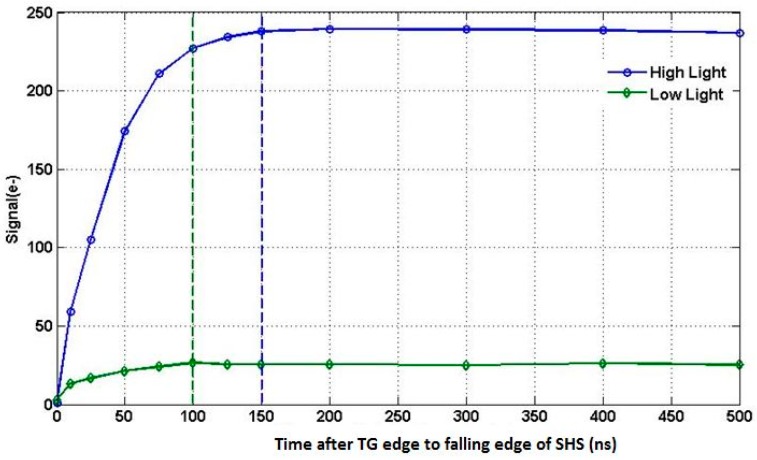
ADC output signal *vs.* time from edge of TG to falling edge of SHS.

Figure 9 below shows the measured ADC output *vs.* TG rising edge to SHS falling edge time for two signal levels when using TG compensation timing. The settling time is 150 ns for a signal of 230 e^−^ and 100 ns for a signal level of 25 e^−^. The 150 ns settling time is less than the dark settling time of 280 ns for conventional timing. The measured settling times are in reasonable agreement with simulation results of 86 ns and 137 ns, respectively.

These simulation results include the calculation of the number of settling time constants (*N*τ) that are required for small and quantized signals. For conventional CIS readout where it is required to convert the maximum signal level to n-bits, the required number of setting time constants is given by Equation (3):

(3) $$N\tau =\text{ ln }(2*\left(1-\text{slewp}\right)*\left(V_{\text{max}}/V_{\text{lsb}}\right)$$

where *V*_max_ is the full signal swing, *V*_lsb_ is the lsb voltage and slewp is the slew percentage of the full signal swing. Assuming a signal swing of 500 mV to 1 V, a slew percentage of 70% and a 12 bit ADC for a conventional CIS device, this would yield *N*τ of 7–9 for conventional CIS devices. For photon counting devices the Vmax is only a few electrons (e.g., 1–20 e^−^). In this case *N*τ will be 0.7 to 3.7.

The *t*_CDS_ is the time between the falling edge of the SHR signal to the falling edge of the SHS signal as shown in Figure 6 and Figure 7. With TG compensation timing and attendant reduced settling time, the *t*_CDS_ can be reduced from the baseline time of 750 ns. In addition to *t*_CDS_, the SF load current is also programmable. The SF load current (Ibias) was adjusted to change the τD of the readout. In conventional CIS, reduced BW can preclude readout of a full signal swing, but can be used with a photon counting CIS as previously discussed. CDS times of 750, 250, 100 and 50 ns were implemented with SF Ibias values of 8.0, 0.8 and 0.4 μA.

100 dark frames were captured for each operating condition, at room temperature with an integration time of 16.5 μs. The frame rate was 56 frames-per-second (frame time of 17.8 ms). Total sensor temporal noise was measured at the ADC output in the dark as a function of *t*_CDS_ and SF Ibias with TG compensation timing implemented. Since the total sensor read noise was measured at the ADC output, this included the SF, PGA and ADC read noise. Noise measurements were then made at the ADC output by overlapping the SHR and SHS pulses during readout to determine the read noise of only the PGA and ADC. This is referred to as base noise in the rest of the paper. The SF noise was then calculated by an rms subtraction of the base noise from the total noise (Equation (4) below):

(4) $$\sigma_{sf}=sqrt(\sigma_{tot}{}^{2}-\sigma_{base}{}^{2})$$

## 3. Read Noise Results

### 3.1. Read Noise Histograms and Average Read Noise

**Figure 10:**
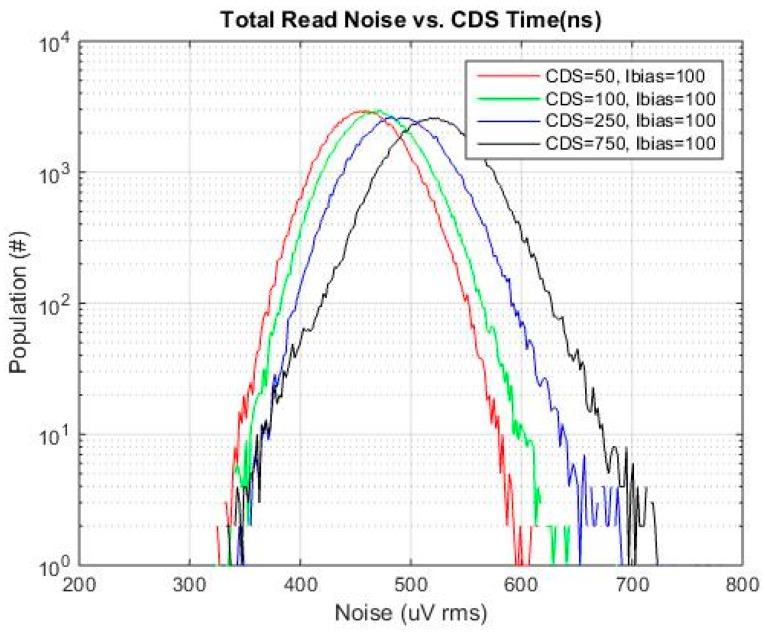
Total read noise for each *t*_CDS_ and SF Ibias of 8 μA (100 μA master current).

Half of one of the imaging arrays was used since this contained the baseline 4T pixel. The data from one bank of ADCs was used to avoid any differences in noise related to layout, routing or timing skew details of the two banks. A histogram of total read noise *vs.* *t*_CDS_ is shown in Figure 10. The Ibias value shown in the legend of the graph is the master Ibias current. The source follower load current is supplied through a current mirror with a reduction ratio of 12.5 (*i.e.*, 100 μA master current is 8 μA source follower load current). The baseline *t*_CDS_ and SF Ibias were 750 ns and 8 μA, respectively.

**Figure 11:**
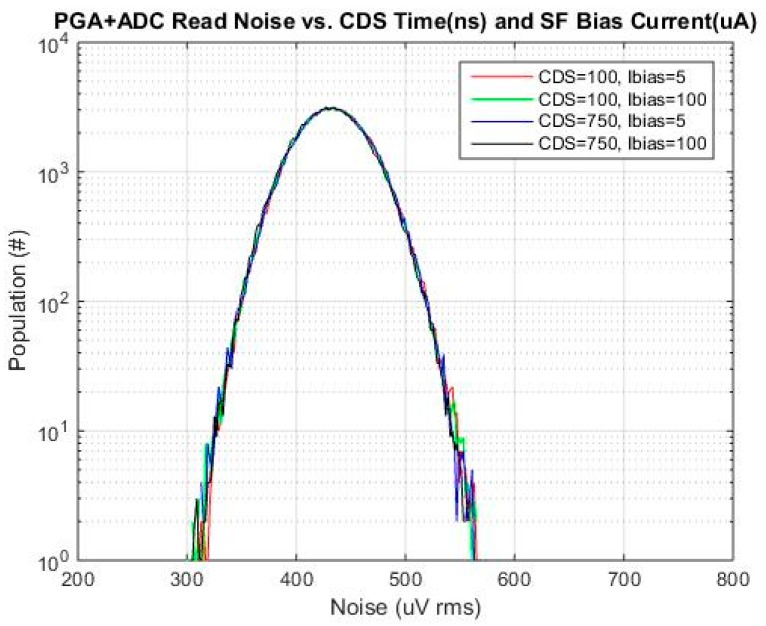
Base read noise histogram for selected *t*_CDS_ and SF Ibias.

A histogram of base read noise *vs.* *t*_CDS_ and SF Ibias is shown in Figure 11. As expected *t*_CDS_ and SF Ibias do not have an effect on the PGA + ADC read noise, and base read noise distribution is Gaussian.

Referring to Figure 10, at the baseline condition of 750 ns, a tail in the histogram is clearly evident. This tail is due to the pixel source follower given this tail is not evident in the base read noise histogram. Such a tail is typical and is attributed to pixels with higher 1/f and RTS noise [14]. As the CDS time is reduced, the tail of the distribution is also reduced. This general trend is expected since the reduced CDS time will reject low frequency 1/f noise, [13,15]. The specific results that are obtained are dependent on the specific thermal noise and 1/f noise magnitude, and specific 1/f noise characteristics of the sensor, [13]. This will be foundry and process specific.

**Figure 12:**
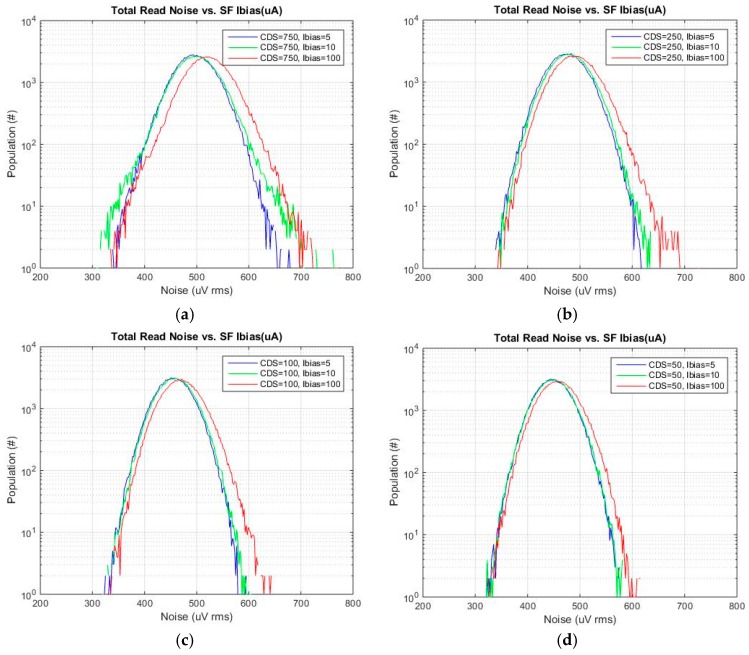
Total read noise histograms for each CDS time *vs.* SF Ibias. *t*_CDS_: (**a**) 750 ns, (**b**) 250 ns, (**c**) 100 ns and (**d**) 50 ns.

Figure 12a–d are total noise histograms for *t*_CDS_ of 750, 250, 100 and 50 ns each with SF Ibias of 8 μA, 0.8 μA and 0.4 μA. For each *t*_CDS_, the total noise is reduced as SF Ibias is reduced from 8 μA to 0.8 μA. There is not much of a change as the SF Ibias is reduced from 0.8 μA to 0.4 μA.

**Figure 13:**
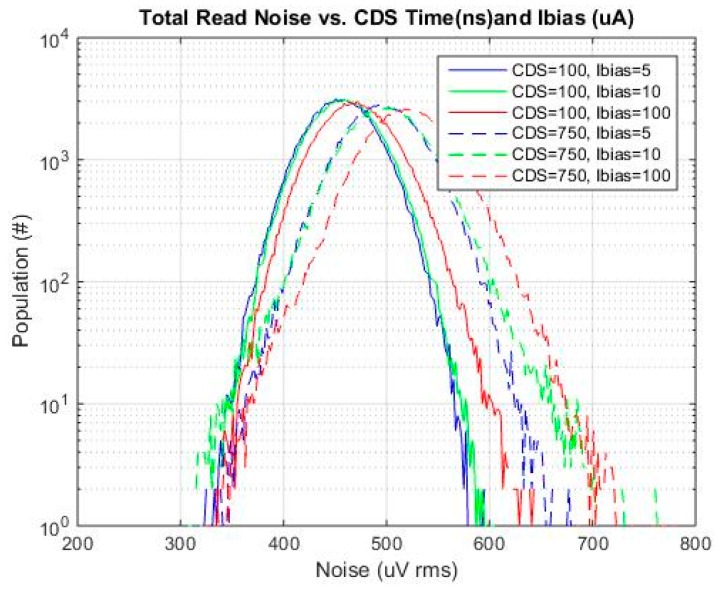
Total read noise histogram for selected CDS times and SF Ibias.

Figure 13 is a histogram of total noise for selected *t*_CDS_ and SF Ibias. Based on measurements and circuit simulations, 100 ns *t*_CDS_ and SF Ibias current of 0.8 μA was selected as a practical minimum operating condition to be able to handle a signal swing of 2 e^−^ (simulated to be 95 ns).

A summary of the average SF read noise vs bias condition is shown in Table 2 below.

This SF read noise was calculated by an rms subtraction of the average base noise from the average total read noise. The average read SF read noise is reduced by a factor of 3.1 (from 246 μV_rms_ to 79 μV_rms_; or 3.2 e^−^_rms_ to 1.0 e^−^_rms_), for the baseline condition of 750 ns and 8 μA compared to 100 ns and 0.8 μA.

These results are compared to the expected reduction in 1/f and thermal noise based on reduction of *τ*D and *t*_CDS_, [15], and based on the baseline thermal and 1/f noise components provided by the process design kit (PDK) for our test sensor. The expected results are shown Table 3 below.

There is reasonable agreement with most of operating conditions, and very good agreement with the noise reduction factor observed from the 100 ns and 0.40 μA compared to the baseline value. An exact agreement would not be likely since the analysis in [15] assumes all 1/f noise has the same slope, and not all of the pixels in the tail of the histogram of our sensor are known to have, nor likely will have identical 1/f noise behavior [13]. A transient noise simulation was also performed for the sensor with the standard noise models provided with the PDK of the 65 nm CIS process. These simulation results predicted SF read noise of 200 μV_rms_ for 750 ns, 8 μA operating point and 85 μV_rms_ for 100 ns, 0.8 μA operating point. This is also in reasonable agreement with the observed results.

### 3.2. Investigation of Individual Pixels vs. CDS Time and SF Ibias

**Figure 14:**
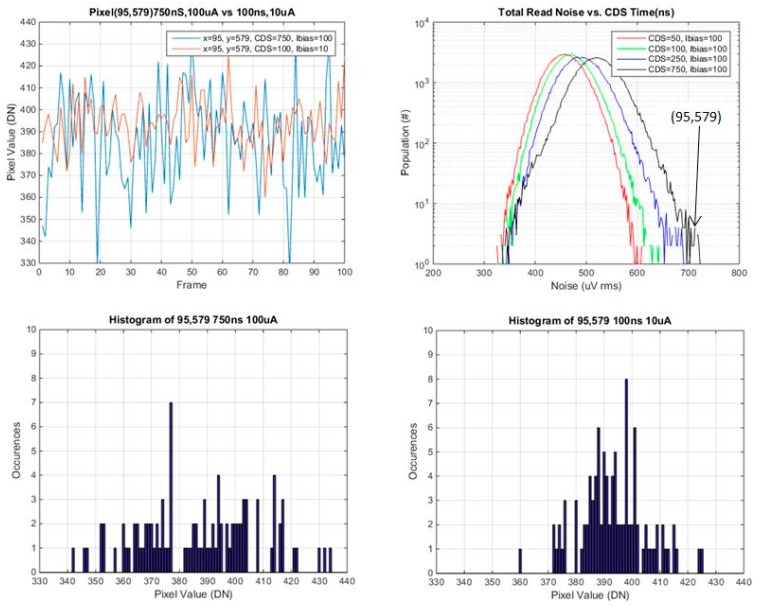
Pixel value *vs.* frame, and histogram of pixel values for pixel 95,579 (from the tail of the noise histogram).

**Figure 15:**
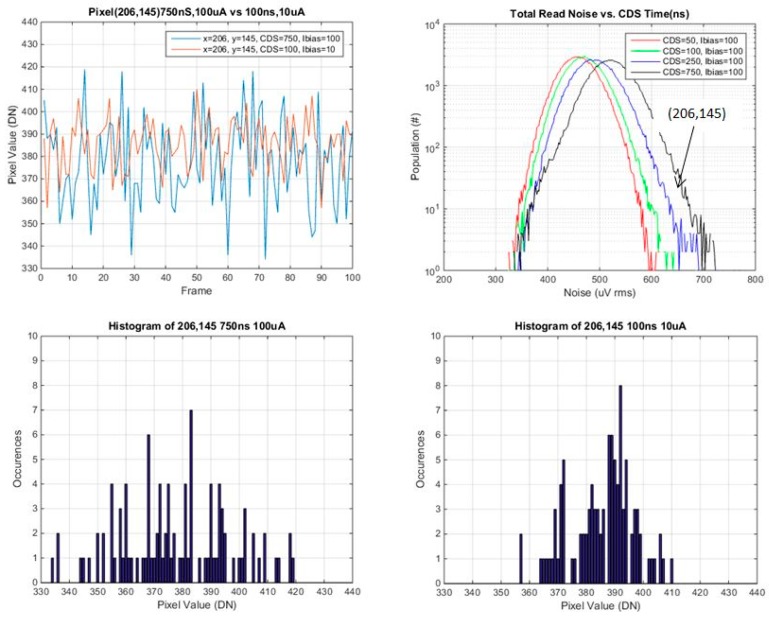
Pixel value *vs.* frame, and histogram of pixel values for pixel 206,145 (from the shoulder of the noise histogram).

**Figure 16:**
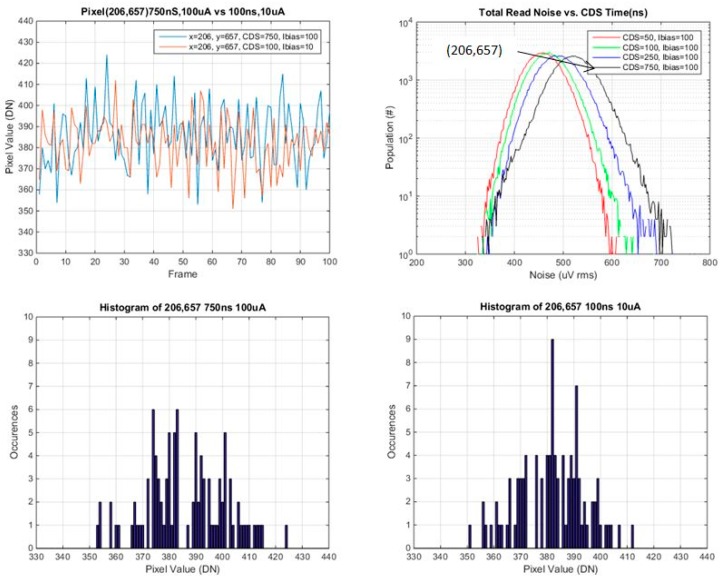
Pixel value *vs.* frame, and histogram of pixel values for pixel 206,765 (from the mode of the noise histogram).

**Figure 17:**
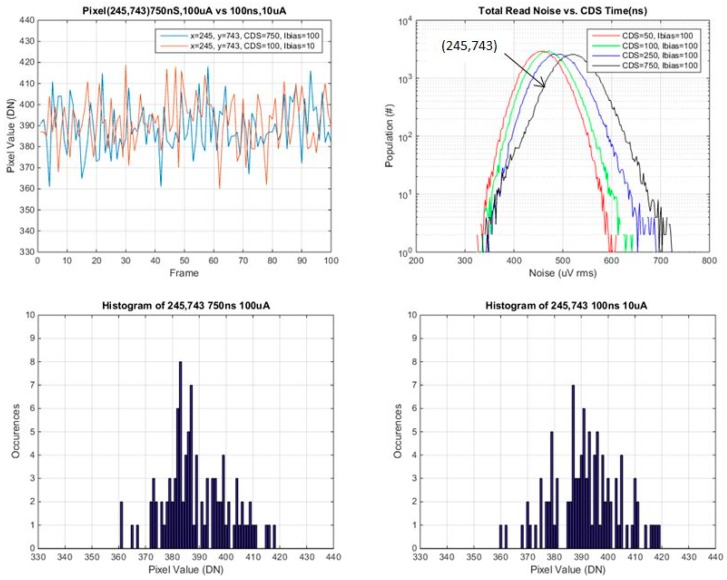
Pixel value *vs.* frame, and histogram of pixel values for pixel 245,743 (from below the mode of the noise histogram).

Several pixels were selected from various points on the read noise histogram, the mode, the tail and selected points in between. Plots of pixel value *vs.* frame #, and histograms of pixel values for the 100 frames, are provided for each of these selected pixels. These are shown in Figure 14, Figure 15, Figure 16 and Figure 17 below.

In general the histograms appear not to be multi-modal which would be indicative of RTS pixels [16], although 100 samples may not be enough in order to see this behavior. It is evident that from comparing histograms of higher noise pixels at 750 ns *t*_CDS_ and 8 μA Ibias *vs.* 100 ns *t*_CDS_ and 0.8 μA Ibias, that the histogram is a significantly tighter distribution for 100 ns CDS time and 0.8 μA Ibias. For lower noise pixels near to or less than the mean of the baseline distribution, there is little or no change in the histograms of the two operating conditions. The low noise histograms may likely appear to be more Gaussian if more samples were taken.

A summary of the ratio of total noise reduction is provided in Table 4 below. Since only 100 samples were used, only general trends can be observed. For a high noise pixel from the tail of the baseline distribution, there is close to a factor of 2 reduction in the total read noise. For pixels near or below the mean there is little or no change in the ratio of the total read noise.

In order to examine this further, the base noise for each pixel was averaged over the 400 frames captured for the baseline noise measurement (100 frames each for *t*_CDS_ of 750 ns and 100 ns and Ibias of 8 μA and 0.4 μA). An rms subtraction of the average base noise from the average total noise for each selected pixel was done to determine the SF read noise for each of the selected pixels. This result is shown in Table 5.

The results from Table 3 are now as expected given pixels in the tail and shoulder of the histogram have high SF read noise and in general will therefore have higher 1/f noise, and will be impacted more by reduced *t*_CDS_ and SF Ibias [13,15]. In contrast pixels 206,657 and 245,743 have low SF read noise, and are likely dominated by thermal noise, and as a result will not change much with reduced *t*_CDS_ and SF Ibias, and may increase slightly due to the reduced SF g_m_ at lower SF Ibias. 

## 4. Discussion

The maximum voltage swing for readout is much lower for a photon counting CIS device than that of a conventional CIS device. As a result the CDS time and dominant time constant of the SF readout can be reduced significantly. We have experimentally shown that reduced CDS time and dominant time constant of the SF readout can provide significant read noise reduction. The ratio of noise reduction will depend on the baseline characteristics of the CIS device. We achieved a factor of three reduction in the average SF read noise for the device used in this study (246 μV_rms_ to 79 μV_rms_). This was a reduction in input referred SF read noise from 3.2 e^−^ to 1.0 e^−^ (CG of 75.6 μV/e^−^). This was is reasonable agreement with the transient noise simulation results completed using the PDK and noise models provided by the foundry, 200 μV_rms_ to 85 μV_rms_.

The conversion gain for the sensor in this study was not optimized, and in general the 1 × 4 shared pixel architecture will have a lower conversion gain than an unshared or 2 × 2 shared pixel. For unshared or 2 × 2 shared pixel architectures, conversion gains in the range of 100 μV/e^−^ to 300 μV/e^−^ have been reported, [8,11,17]. For a device with similar SF device characteristics, but a higher conversion gain (e.g., 200 μV/e^−^), the reduced *t*_CDS_ and SF Ibias would provide a SF input referred read noise of 0.38 e^−^. In addition the SF noise for this sensor (200 μV_rms_ to 250 μV_rms_), was high by state of the art standards (<100 μV_rms_).

Further work is planned to look at noise characteristics of individual pixels and the subsequent effects of reduced *t*_CDS_ and SF BW in more detail. For our test sensor, this will require many more frames (1000–1500) in order to have sufficient statistical accuracy in the rms subtraction of total read noise from base read noise.

## Figures and Tables

**Table 1:** Sub 1 e^−^ SF read noise results from various references.

Ref #	Noise Reduction Approach	Conversion Gain (μV/e^-^)	Analog Gain	Number of Reads	Read Noise (μV rms)	Read Noise (e^-^ rms)	Pixel Size (μm)	Process Node (nm)
[6]	CS AmpHigh CG	300	10	1	258	0.86	11	180
[7]	High CG	240		1	120	0.50	5.5	180
[8]	High CG	426		1	137	0.28	1.4	65
[8]	High CG	256		1	97	0.32	1.4	65
[9]	CMS		64	4		0.70^1^	10.0	180
[10]	CMS	110	16	5	73	0.66	1.1	
[11]	Bch SF	185	64	1	74	0.40	7.5	180
[12]	CMSInver. Cycling	~400		1600	136	0.34	25	180

^1^ This reference provided total read noise only (SF read noise was not determined).

**Table 2:** Measured average SF read noise (μVrms) for selected *t*_CDS_ (ns) and SF Ibias (μA).

CDS Time (ns)	Ibias (μA)		
	8	0.8	0.4
750	246	217	205
250	189	160	149
100	105	79	78

**Table 3:** Expected average SF read noise (μV_rms_) as a function of *t*_CDS_ (ns) and SF Ibias (μA).

CDS Time (ns)	Ibias (μA)		
	8	0.8	0.4
750	246	191	179
250	191	148	130
100	157	98	79

**Table 4:** Normalized total noise for selected pixels *vs.* *t*_CDS_ (ns) and SF Ibias (μA).

	CDS time (ns), SF Ibias (μA)			
Pixel (location in histogram)	750, 8.0	250, 8.0	100, 8.0	100, 0.8
95, 579 (tail)	1	0.82	0.67	0.52
206, 145 (shoulder)	1	0.74	0.74	0.57
206, 657 (mean)	1	1.02	0.85	0.82
245, 743 (< mean)	1	1.07	1.07	1.02

**Table 5:** SF read noise for selected pixels *vs.* *t*_CDS_ (ns) and SF Ibias (μA).

	SF noise (μV) rms
Pixel (locationin histogram)	
95, 579 (tail)	187
206, 145 (shoulder)	151
206, 657 (mean)	88
245, 743 (< mean)	62
